# Antioxidant Therapy in Nonalcoholic Steatohepatitis

**DOI:** 10.1155/2012/947575

**Published:** 2012-11-22

**Authors:** Said A. Al-Busafi, Mamatha Bhat, Philip Wong, Peter Ghali, Marc Deschenes

**Affiliations:** ^1^Department of Medicine, College of Medicine and Health Sciences, Sultan Qaboos University, Alkhoudh, Muscat 123, Oman; ^2^Department of Gastroenterology and Hepatology, McGill University Health Centre, Montreal, QC, Canada H3A 1A1

## Abstract

Nonalcoholic steatohepatitis (NASH) affects up to 3% of the North American population. It occurs as a manifestation of the insulin-resistant state and oxidative stress is thought to be a key component of its pathophysiology. Exercise and diet, which are the mainstay of therapy, are difficult to achieve and maintain with a disappointing long-term compliance record. There is growing literature on the potential for antioxidant therapy. The recent literature strongly suggests that vitamin E supplementation and other putative free radical scavengers and/or antioxidants are beneficial in improving biochemical and histological parameters in NASH.

## 1. Introduction

Nonalcoholic fatty liver disease (NAFLD) is a clinico-histopathological entity with histological features that resemble alcohol-induced liver injury; but by definition, it occurs in patients with no recent or ongoing significant alcohol consumption (>21 drinks on average per week in men and >14 drinks on average per week in women) [1]. NAFLD is becoming a greater health concern in North America, with increasing rates of obesity and type II diabetes. An insulin-resistant state and the presence of the metabolic syndrome are strongly associated with NAFLD [2, 3]. Up to 30% of the current North American population has NAFLD, with 10% of them having subclinical hepatic inflammation known as nonalcoholic steatohepatitis (NASH) or fibrosis [4]. NAFLD encompasses a spectrum of conditions, starting with isolated hepatic steatosis, progressing to NASH, cirrhosis, and ultimately hepatocellular carcinoma (HCC) [5]. Moreover, there is a growing body of the literature suggests that HCC might occur in the settings of NASH in the absence of cirrhosis [6]. 

The gold standard of diagnosis is liver biopsy, which has both diagnostic and prognostic value [1]. Macrovesicular steatosis is seen predominantly in zone 3 although it may be panacinar. Hepatocyte ballooning, Mallory bodies, a mixed inflammatory infiltrate, and pericellular fibrosis are additional features typical of NASH [7]. The Brunt classification enables staging of fibrosis in NASH by considering these various histological features [8].

## 2. The Role of Oxidative Stress in the Pathogenesis of NASH 

The pathogenesis of NASH has not been fully elucidated. Currently, the most widely supported theory in the pathogenesis of NASH is a “two-hit” theory. According to this theory, the “first hit” involves fat accumulation in the hepatocytes, where insulin resistance is suggested to be the key pathogenic factor [9, 10]. Insulin resistance, found in obesity and type 2 diabetes, leads to increased lipolysis and increased hepatic uptake of free fatty acids (FFA) with increased hepatic triglyceride synthesis and accumulation. The “first hit” increases the vulnerability of the liver to multiple factors that constitute the “second hit” leading to hepatic injury, inflammation, and fibrosis. Oxidative stress and subsequent lipid peroxidation, proinflammatory cytokines, adipokines, and mitochondrial dysfunction are included among these factors [11]. 

Several studies have demonstrated that oxidative stress is a major player triggering the progression of steatosis to steatohepatitis [12–15]. Mitochondria play a major role in FFA oxidation and are responsible for the majority of disturbances occurring in lipid metabolism. In the process of FFA oxidation, the mitochondria leak reactive oxygen species (ROS) mainly in the form of hydrogen peroxide. In order to prevent oxidative stress, there is a continuous balance between intrahepatic antioxidants (such as glutathione, vitamin E,  *β*-carotene, and vitamin C) and ROS. When there is an imbalance, however, ROS trigger steatohepatitis by lipid peroxidation, cytokine induction, and Fas ligand induction. In the presence of steatosis, mitochondrial ROS oxidize accumulated hepatic fat, causing lipid peroxidation which leads to hepatocytes necrosis and death and increases collagen synthesis, that is, fibrosis. ROS also induce the secretion of cytokines such as tumor necrosis factor gamma (TNF-*γ*), transforming growth factor beta (TGF-*β*) and interleukin-8 (IL-8) which cause hepatocytes death. Interestingly, TGF-*γ* is involved in the formation of Mallory bodies and activating collagen synthesis by hepatic stellate cells inducing fibrosis. IL-8 is a chemoattractant that gives rise to neutrophils infiltration. The third consequence of ROS in this sequence to steatohepatitis is Fas ligand (a membrane receptor) expression by hepatocytes. This membrane receptor enables the hepatocyte to interact with Fas ligand on another hepatocyte resulting in fractional liver cell killing [16]. 

In addition, lipid peroxidation and ROS can lead to depletion of antioxidant enzymes, thus rendering the liver susceptible to oxidative injury [17, 18]. Serum levels of xanthine oxidase, a generator of ROS, are higher in patients with NASH compared with controls, whereas levels of multiple antioxidant enzymes are lower [19]. In addition, the induction of heme oxygenase-1, an antioxidant defense enzyme, interrupted the progression of steatohepatitis by inducing an antioxidant pathway and suppressing proinflammatory cytokines [20]. A correlation between disease severity and increased expression of oxidative scavenger receptors has been described [21].

Impaired inactivation by antioxidant depletion is the proposed rationale for antioxidant supplementation in the treatment of NAFLD. As mentioned above, ROS are the main cause for the development of steatohepatitis and fibrosis. Steatosis-induced lipid peroxidation by ROS consumes antioxidant enzymes, glutathione, and vitamin E. The use of exogenous antioxidants is thought to be able to minimize oxidative stress with NASH. 

## 3. Measures of Oxidative Stress and Antioxidant Defense 

Various measures of oxidative stress and antioxidant status defense have been researched, although none are well developed enough to be incorporated into practice. A study of 22 patients with biopsy-proven NASH versus 22 healthy controls was conducted to compare and contrast oxidative status [22]. Fasting blood samples were obtained, and total serum peroxide levels were measured. The total antioxidant status is a novel assay that was performed using these samples. In this assay, an oxidative reaction is suppressed by antioxidants present in a patient's serum sample, thus preventing the color change of a substrate. The oxidative stress index, defined as the ratio percentage of total peroxide to total antioxidant potential, was also calculated. The total peroxide level and oxidative stress index correlated with fibrosis scores on liver histology (*P* < 0.05). On the other hand, the total antioxidant status was significantly decreased and negatively correlated with fibrosis scores (*P* < 0.05). Other studies have supported the theory of increased oxidative stress in NASH, and a serum index of oxidative stress was shown to independently predict liver fibrosis [23–25].

Additional serum biomarkers of oxidative stress include oxidized LDL (ox-LDL) and thiobarbituric acid-reacting substances (TBARS) [26]. These lipid peroxidation products were found to be significantly higher among patients with NASH as compared to age-, gender- and body mass index-matched controls. In the same study, the total daily intake of vitamin E and carotenoids, known antioxidants, was not significantly different between the NASH subjects and controls. However, the daily intake of these antioxidants by the cohort as a whole was much lower than recommended. A more recent study revealed that NASH patients had much lower levels of serum vitamin E and carotenoids than healthy controls [27]. 

## 4. Current Management of NASH

The goals of therapy in NASH are to slow down its progression to cirrhosis and potentially reverse some damage by recommending lifestyle changes. The emphasis should be on gradual weight loss through caloric restriction and exercise. This leads to an improvement in insulin resistance, liver enzymes, and liver histology [1, 28]. The patient should follow a low-fat, low-glycemic diet, rich in fruits and vegetables. Theoretically, diets rich in vitamin E (*α*- and *γ*-tocopherol), carotenoids (*α*- and *β*-carotenes), ascorbic acid, and monounsaturated fatty acids would have an antioxidant effect.

A variety of pharmacotherapeutic strategies have been attempted in NASH, but most trials have been too short to determine and impact on important patient-centered clinical outcomes [29]. There has recently been a growing evidence for antioxidant therapy in the treatment of NASH, which is the focus of the current discussion. 

## 5. Antioxidant Therapy for NASH

### 5.1. Vitamin E 

#### 5.1.1. Pharmacology of Vitamin E

Vitamin E is a lipophilic antioxidant that protects cell membranes from oxidation and destruction. In 1959, the FDA formally recognized vitamin E as an essential nutrient for human health [30]. In nature, vitamin E is found in a variety of foods including green leafy vegetables oils, meat, and eggs [31]. Dietary vitamin E content is variable as it is proportional to vegetable oil intake. The United States National Academy of Sciences Food and Nutrition Board recommends 15 mg (22.4 IU) of dietary *α*-tocopherol per day [32]. 

The role of vitamin E in the treatment of NASH is based on its activity as a free radical scavenger. Vitamin E is a chain-breaking antioxidant in free radical reactions, which is an important step in lipid peroxidation and membrane stabilization [33]. Vitamin E acts as a regulator of the activity of genes such as TGF-*β*1, peroxisome proliferator-activated receptors and genes regulating apoptosis, inflammation and collagen deposition [30]. In addition, vitamin E tocotrienols improved insulin sensitivity in animal study through activating peroxisome proliferator-activated receptors [34]. Other functions of vitamin E include inhibition of cell proliferation, platelet aggregation, and monocyte adhesion [35].

#### 5.1.2. Evidence behind Vitamin E

Animal studies have shown that vitamin E improves fibrosis, reduces mitochondrial lipid peroxidation, and corrects oxidative stress in animal models of liver disease associated with oxidative injury [36]. In rat models of NASH, vitamin E inhibited TGF-*β*1 gene expression, resulting in amelioration of liver necrosis and fibrosis [37]. Moreover, a recent study provided the first evidence that vitamin E can prevent the development of NAFLD, largely by ameliorating oxidative stress, necroinflammation, and hepatic apoptosis [38]. In addition, vitamin E has been shown to reduce oxidative damage and tissue inflammatory mediator TNF-*α* levels, while reducing PPAR-*γ* expression [39]. 

These observations in animal studies provided a rationale for evaluating the use of vitamin E in patients with NASH (Table 1). In addition, a reduced daily dietary intake of vitamin E [51] as well as reduction of its serum levels [52] has been documented in patients with NASH. 

An initial observational trial demonstrated normalization of aminotransferases in 11 obese children with NASH following 4 to 10 months of vitamin E (400–1200 IU daily) [50]. A second observational study of liver biopsy-proven NASH in adults treated with vitamin E (300 mg/daily) for 1 year demonstrated improved steatosis and concomitant decrease in TGF-*β*1 levels [49]. This led the way to subsequent randomized placebo-controlled trials of vitamin E. A study of 28 children with obesity-related NASH (14 treated with low-calorie diet and 14 with vitamin E, 400 mg for 2 months and 100 mg for 3 months) [46], and another study of 16 biopsy-proven NASH patients treated with vitamin E (800 IU/daily) [48], were not able to demonstrate any benefit from vitamin E treatment. However, a subgroup analysis showed that vitamin E therapy improved aminotransferase levels in patients who were noncompliant with diet, even when weight loss was not achieved, which suggests that vitamin E may be useful in obese children who are unable to adhere to a low-calorie diet [46]. 

Despite these varying results, other studies have demonstrated a reduction in oxidative stress and cytokine markers in NASH patients treated with vitamin E, supporting the role of vitamin E as an antioxidant. An uncontrolled pilot study of 10 patients with NASH showed improvement in aminotransferase levels along with decreased plasma levels of thioredoxin and thiobarbituric acid reactive substance, indicators of oxidative stress, after 6 months of 300 mg vitamin E daily [44]. 

Since NASH has a multilevel and a multifactorial pathogenesis, it is logical to hypothesize that a drug combination might be a more appropriate therapeutic strategy. In a pilot study, Sanyal et al. suggested that the combination of vitamin E and pioglitazone, an insulin sensitizer, was superior to vitamin E alone for improving liver histology [45]. Vitamin E was also able to increase the effect of ursodeoxycholic acid in normalizing ALT among patients with histologically confirmed NAFLD [43, 53]. There have been attempts to combine vitamin C as a potent antioxidant along with vitamin E. Harrison et al. randomized 45 NASH patients to receive vitamin E plus vitamin C (1000 IU/1000 mg/day) versus placebo for 6 months and found that patients receiving the combination treatment demonstrated an improvement in fibrosis but not necroinflammatory activity or serum ALT levels [47]. More recently, Foster et al. published a randomized study of atorvastatin and antioxidants (vitamins E and C) versus placebo for 4 years which demonstrated an improvement in liver steatosis based on computed tomography scans [42]. 

All of the aforementioned studies had provided some evidence of a benefit of vitamin E in NASH; however, this evidence was limited, as the sample sizes were small and studies were performed at single centers. Moreover, vitamin E was administered in combination with other medications and not as monotherapy. In addition, the dose of vitamin E used in these trials as well as subsequent ones is very high comparing to FDA daily recommendation, that is, 15 mg (22.4 IU) per day. More recently, two larger RCTs (PIVENS [40] and TONIC [54]) comparing vitamin E versus placebo for the treatment of patients with NASH with histology as an end point were published. Fifty-seven pediatric NASH patients were treated with vitamin E (800 IU/day) for 96 weeks. Vitamin E was superior to placebo in improving ballooning and NASH activity score, but no effects on liver fibrosis or portal, and lobular inflammation were noted [54]. In the second study, Sanyal et al. treated 84 nondiabetic noncirrhotic NASH patients with vitamin E (800 IU/day) for 96 weeks, demonstrating that this treatment, compared with placebo, was associated with a significant improvement in serum aminotransferase levels, hepatic steatosis, and lobular inflammation, but not portal inflammation and hepatic fibrosis [40].

In summary, the potential benefits of a combination treatment have been demonstrated in many of these studies. The two most recent RCTs seem to indicate that vitamin E monotherapy, at least in the early stage of the disease, improves serum aminotransferase levels and some histological features of NASH [40, 50]. However, there is no evidence that vitamin E improves more advanced histologic features such as portal inflammation and liver fibrosis, which are linked with progression of the disease. In addition, these studies have no followup and, therefore, the effects of treatment on long-term morbidity and mortality are unknown. Moreover, the effects of vitamin E in NASH patients with diabetes and significant fibrosis or cirrhosis have not been assessed [49].

A recent meta-analysis showed that RCTs with vitamin E and other antioxidants were heterogeneous with respect to type and dose of drug, treatment duration and followup, population (pediatric versus adult), and implementation of lifestyle intervention, thus yielding conflicting results [55]. This extreme heterogeneity prevents any firm conclusion on the effect of vitamin E on NAFLD. 

### 5.2. Safety of Vitamin E

Although RCTs done in NASH population have not reported serious side effects related to vitamin E, it is important to emphasize that none of these trials was powered to evaluate adverse events. A meta-analysis of controlled clinical trials of vitamin E supplementation revealed no significant effect on all-cause mortality across all trials, although high-dose supplementation (≥400 IU daily) did increase it [56]. However, the slightly increased risk in mortality may only be applicable to patients with heart disease, especially given that that vitamin E is associated with blunted efficacy of statin drugs [57]. 

A recent large RCT, a mean followup of 8 years, showed that vitamin E (400 IU every other day) had no significant effect on total mortality but was associated with an increased risk of hemorrhagic stroke [58]. Moreover, another recent RCT showed that vitamin E administered at a dose of 400 IU/day increased the risk of prostate cancer in relatively healthy men [59].

In summary, while some concern has been raised about potentially increased bleeding risk and all-cause mortality with vitamin E treatment in other conditions, it is unclear whether these findings are attributable solely to vitamin E intake. It would be helpful to examine its safety in a larger number of patients, for longer treatment durations and perhaps in higher doses or in combination with other agents. 

Despite these limitations and, in summary, in the recent AASLD guidelines, vitamin E administered at daily dose of 800 IU/day is considered as a first line therapy for nondiabetic adults with biopsy-proven NASH [60]. In addition, vitamin E is not recommended to treat the following groups till more supporting data become available: NASH in diabetic patients, NAFLD without liver biopsy, NASH cirrhosis, or cryptogenic cirrhosis. 

### 5.3. Other Antioxidants

Several other antioxidants have been evaluated in single observational studies and make it difficult to conclude any firm therapeutic recommendations.

N-acetylcysteine (NAC) is a precursor of glutathione, the most powerful antioxidant in the liver. An uncontrolled study of 11 NASH patients treated with NAC revealed significantly improved aminotransferase levels [61]. A randomized controlled trial of 35 patients of NAC versus no therapy over a 4-week period showed nonstatistically significant improvements in the aminotransferases [62]. The combination of NAC and metformin improved liver appearance and reduced fibrosis in patients with NAFLD [63].

NASH develops as the result of “second hits” and one of those “hits” is the steady depletion of SAMe [64]. This has led to interest in using SAMe to prevent NASH from developing in people who already have NAFLD [65]. S-Adenosylmethionine (SAMe) replenished glutathione levels, providing protection against liver injury in a rat model [66]. Studies using drugs that increase SAMe levels are known to reduce severity of NAFLD [67, 68]. 

Betaine, an agent known to restore glutathione stores, was shown to significantly decrease transaminitis, hepatic steatosis, and even the stage of fibrosis in an observational study of 7 patients over a 1-year period [48, 68]. An 8-week RCT of 191 NASH patients on betaine versus placebo also revealed a reduction in transaminitis and hepatic steatosis [69]. On the other hand, a more recent RCT including 55 patients with biopsy proven NASH was unable to demonstrate any improvement in liver enzymes or histology compared with placebo [70]. 

Probucol is a novel antihyperlipidemic agent with powerful antioxidant activity, preventing lipid oxidation. It showed promise through significant reduction in aminotransferase levels and improvement in liver histology in a small observational study of 8 patients [71]. An RCT showed probucol to be significantly effective in decreasing the ALT levels in patients with NASH [72]. However, probucol concomitantly decreased HDL levels, which is of concern in patients with coronary artery disease.

Viusid, a nutritional supplement incorporating various antioxidants, was evaluated in an RCT of biopsy-proven NAFLD patients [73]. Sixty patients were randomized to Viusid along with hypocaloric diet and aerobic exercise versus hypocaloric diet and exercise alone over a 6-month period. Diet and exercise on their own improved hepatic steatosis and steatohepatitis; however, Viusid further synergistically improved these histologic parameters.

Silibinin (milk thistle), a free radical scavenger with antifibrotic properties, alleviated steatohepatitis and NASH-induced lipid peroxidation when given over 12 weeks in a rat model of NASH [74]. A pilot study in patients with NAFLD found improvement in liver enzymes and insulin resistance, when silibinin was given with vitamin E and phospholipids [75].

None of the aforementioned other antioxidants have substantive evidence of efficacy in NASH; we are thus unable to make definitive recommendations on their use.

## 6. Conclusion

The role of oxidative stress in the pathogenesis of NASH has been increasingly recognized in recent years and has resulted in greater attention being paid to antioxidant therapy. Lifestyle measures and treatment of risk factors do improve NASH; however, these are difficult to achieve and maintain with disappointing long-term compliance record. Thus, the prevalence of this disease and its consequences in the Western population merit greater attention to pharmacotherapy beyond insulin sensitizers. There is strong and growing evidence for vitamin E. The results of RCTs published so far are promising but new trials with histological end points are needed to define the efficacy and safety of vitamin E in the treatment of NASH. In addition, in these trials, a precise identification of patients with regard to NASH risk factors, pathogenesis, and prognosis will help identifying the best therapeutic strategies tailored for each NASH patient. Furthermore, the new trials should also focus on assessing the potential mechanisms of action of vitamin E in NASH patients. While waiting for these trials and based on the recent AASLD guidelines, we do recommend using vitamin E (800 IU/day) for nondiabetic adults with biopsy proven NASH in addition to lifestyle changes and risk factors control.

## Figures and Tables

**Table 1:** Published studies on the effects of vitamin E in patients with NAFLD.

Author	Year	Number	Intervention	Design	Durations (months)	Liver enzyme	Outcomes Fatty liver by imaging	Histology
Lavine et al. [41]	2011	57	Vitamin E versus metformin versus Placebo	RCT	24	NA	NA	Improved ballooning and NASH activity, no effect on lobular inflammation and fibrosis

Foster et al. [42]	2011		Atorvastatin and antioxidants (vitamins E + C) versus Placebo	RCT	48	NA	Improved steatosis on CT scans	NA

Sanyal et al. [40]	2010	247	Vitamin E versus pioglitazone versus placebo	RC T	24	Improved	NA	Improved steatosis and Inflammation (NS versus pioglitazone)

Dufour et al. [43]	2006	48	Vitamin E + UDCA versus UDCA + placebo versus placebo + placebo	RCT	24	Yes	NA	Steatosis improved

Kawanaka et al. [44]	2004	10	Vitamin E	Open label	6	Yes	NA	NA

Sanyal et al. [45]	2004	10	Vitamin E versus vitamin E + pioglitazone	RCT	6	Yes	NA	Yes

Vajro et al. [46]	2004	28	Vitamin E versus low calorie diet	RCT	5	Yes but NS	NA	No

Harrison et al. [47]	2003	49	Vitamin C + vitamin E + weight loss counselling	RCT	6	No	NA	Yes (fibrosis) No (inflammation/necrosis)

Kugelmas et al. [48]	2003	16	Vitamin E	RCT	3	Yes (NS)	NA	NA

Hasegawa et al. [49]	2001	12	Vitamin E	OL	12	Yes	NA	Yes

Lavine [50]	2000	11	Vitamin E	OL	4–10	Yes	NA	NA

N/A: not available; RCT: randomized controlled trial; OL: open label; NS: nonsignificant.
